# A Soft Coprocessor Approach for Developing Image and Video Processing Applications on FPGAs

**DOI:** 10.3390/jimaging8020042

**Published:** 2022-02-11

**Authors:** Tiantai Deng, Danny Crookes, Roger Woods, Fahad Siddiqui

**Affiliations:** 1 Department of Electronics and Electrical Engineering, The University of Sheffield, Sheffield S1 3JD, UK; t.deng@sheffield.ac.uk; 2 School of Electronics, Electrical Engineering and Computer Science, Queen’s University Belfast, Belfast BT7 1NN, UK; r.woods@qub.ac.uk (R.W.); f.siddiqui@qub.ac.uk (F.S.)

**Keywords:** image processing, FPGA, soft coprocessor, soft processor, image algebra

## Abstract

Developing Field Programmable Gate Array (FPGA)-based applications is typically a slow and multi-skilled task. Research in tools to support application development has gradually reached a higher level. This paper describes an approach which aims to further raise the level at which an application developer works in developing FPGA-based implementations of image and video processing applications. The starting concept is a system of streamed soft coprocessors. We present a set of soft coprocessors which implement some of the key abstractions of Image Algebra. Our soft coprocessors are designed for easy chaining, and allow users to describe their application as a dataflow graph. A prototype implementation of a development environment, called SCoPeS, is presented. An application can be modified even during execution without requiring re-synthesis. The paper concludes with performance and resource utilization results for different implementations of a sample algorithm. We conclude that the soft coprocessor approach has the potential to deliver better performance than the soft processor approach, and can improve programmability over dedicated HDL cores for domain-specific applications while achieving competitive real time performance and utilization.

## 1. Introduction

Image processing algorithms are used in many applications, including image classification, medical image processing, video surveillance and target detection and tracking [1,2,3]. These applications have been embedded in more and more devices such as smartphones, unmanned autonomous vehicles and surveillance cameras [4,5,6]. Safety critical image processing applications require the processing system to be accurate, and often fast [7]. With the rapid development of image sensors, the resolution of images and videos is becoming higher than ever. For high-resolution images, traditional processors struggle to keep up with increasing resolutions [8]. It may not be possible to process very large images in real-time using conventional CPUs. Thus, it is necessary to consider ways of accelerating the most time-consuming computing tasks of the application in these cases. Commonly, there are four approaches to accelerating image processing algorithms, namely: multi-core clusters of CPUs, GPUs, FPGAs and ASICs. CPUs and GPUs are instruction-based processors, and so they operate on the normal fetch-execute cycle model. This means that it can take several clock cycles to execute one instruction. They are also relatively high power compared to ASICs and FPGAs when implementing the same application [9]. ASICs usually have the best performance and lowest power, but they are not programmable and are very expensive to produce. FPGAs are somewhere between GPUs and ASICs. They are capable of producing low power, low cost but high-performance solutions. However, the design time for custom cores can be much longer than for GPUs [10]. In the field of image processing, because of the independence of pixels, FPGAs can produce good speedup, particularly when used as a coprocessor for low-level image processing operations [11]. However, the key challenge is to speed up the process of producing an FPGA-based solution to image processing application problems.

The need to accelerate the application development process is generally acknowledged. Although vendors and researchers have been putting effort into creating higher-level design environments for building hardware accelerators using FPGAs, some problems still remain [10,12,13,14]. Hardware designers tend to use ‘high-level’ in the sense that the syntax is at a higher level than Very high-speed integrated circuit Hardware Description Language (VHDL) or Verilog HDL [15]. However, for application developers and software programmers, high-level means that hardware design issues can be practically ignored, and the coding focuses on the application alone, as though the developer were coding for a PC. To application developers, the above tools remain low level, even if they use C syntax [15]. If application developers use the tools naively, without taking hardware design issues into account, very inefficient hardware is likely to result. Furthermore, although these High-level Synthesis (HLS) tools are described as high-level, there are some features of the input language that cannot be synthesized. For example, Xilinx Vitis HLS does not support the use of pointers and dynamic memory allocation in C [13,16].

Since the result of the HLS tools above is still HDL, users typically require the usual long re-synthesis time when they make changes to the algorithm or application [14]. This hinders the experimental nature of image processing application development, which is one of the targets of this paper.

Xilinx recently aimed to shorten the synthesis time by their new product, Adaptive Computing Accelerating Platform (ACAP) and released an early product of the ACAP family, Versal [17]. The main advantage of the ACAP family is its ability to rapidly perform re-synthesis (within milliseconds). Xilinx also provides its AI Engine to accelerate the deployment of AI applications on selected Xilinx devices. Combined with Xilinx Vitis, the development of AI and image processing applications on some Xilinx devices can be significantly accelerated [18]. Unfortunately, only some of the latest Xilinx devices support this feature.

Thus, the current challenges to using FPGAs to accelerate an image processing system can be summarized as follows:

1. It is hard to achieve both programmability and performance on FPGAs across all devices.
2. Current vendors’ HLS tools still require users to be knowledgeable about hardware and the limitations of the tools.
3. Lengthy synthesis time is a hindrance during experimental and iterative image processing system development.

In this paper, a higher-level approach for image processing system development is proposed to address to some extent the above-mentioned challenges. We will present a number of concepts, which are integrated into a prototype Soft Coprocessor System (SCoPeS), to support the development of FPGA-based image processing applications. Detailed contributions of the paper are as follows:

1. We propose the concept of customizable Soft Coprocessors (SCPs) as the basic building block for stream-based applications. We allow users to chain SCPs together so they can communicate directly with each other and not merely with the host. We use AXI4-Stream Interconnect to connect the SCPs in a system. In this way, we provide users with a flexible system that can be programmed as a Data Flow Graph (DFG). Users do not normally need to re-synthesize when they change the DFG.
2. We provide a set of customizable Software Coprocessors based on the key concepts of Image Algebra (IA), including a range of point, neighborhood, and global operations.
3. We provide a set of efficient hardware skeletons for defining new IA-like operations, where users need only supply their own C-based pixel-level function. This enables the creation of very efficient function-specific SCPs.

Our prototype SCoPeS environment includes several tools to support the SCP approach. A hardware configuration generator tool enables users to specify the number of each type of SCP to be available for the current application project. Provided the application uses only this pool of SCPs, no resynthesis is required. A code generator enables users to define their applications in terms of a text-based DFG. (This is in place of special tools for editing a graphical view of the DFG). Users can edit the textual DFG description, normally without requiring re-synthesis.

The rest of this paper is organized as follows. In Section 2, we introduce the background and related work in terms of Image Algebra, high-level programming models and different FPGA implementations of image processing algorithms and systems. In Section 3, we provide a user’s view of our design approach. In Section 4, we describe the architectures and underlying implementation, including our Generic and Function-specific Image Algebra-based SCPs and how they connect and communicate with each other. In Section 5, we demonstrate how we create a new image processing system using our SCoPeS environment. In Section 6, a comparison between different design approaches for a simple image processing operation is presented for evaluation purposes.

## 2. Background and Related Work

### 2.1. Current Tools for Designing FPGA Custom Cores in a High-Level Environment

Modern FPGAs are no longer thought of as arrays of gates, but as collections of larger-scale functional blocks, integrated using programmable logic. They are still programmable but are not restricted to programmable logic (PL), and sometimes come equipped with on-chip ARM processors or embedded GPUs. When implementing an image processing system on FPGAs, the design effort is a critical project requirement. Very large image processing systems are difficult to design efficiently and require very detailed hardware knowledge. To address this challenge, vendors have released their HLS tools to reduce the design time. The syntax of design description languages has moved up from VHDL/Verilog HDL to C/C++ level because of HLS tools such as Vivado HLS and Intel HLS compilers [19,20]. Applications are becoming more complex. System-on-chip solutions are achievable through the hybrid architecture of ARM+PL and the HLS design approach.

There are also some HLS tools from academia, such as LegUp [21], CyberWorkBench [22], autoBridge [23] and LeFlow [24]. autoBridge is used specifically for floor planning and pipelining high-frequency designs on multi-die FPGAs. LeFlow is designed specifically for deep learning inference implementation. LegUp can generate a hybrid system of custom cores and soft processors; the other tools only generate custom cores. With currently available HLS tools, users need to rely on the vendor’s tools to integrate the RTL design into a whole system, which is a non-trivial task. After the HLS stage, there is generally no additional help for users to integrate their resulting system.

### 2.2. Soft Processors

As an alternative to the inflexible custom core approach, it has become popular to provide cores for simple programmable processors. These allow users to program in high-level languages. A soft processor is achieved by configuring FPGA hardware resources as a processor. Soft processors can reduce the design time through using a high-level language. They also reduce the hardware knowledge required to design a full system. However, single core performance of a soft processor is usually poor, since soft processors go through the standard fetch-execute cycle for each instruction, and they cannot run at as high a clock rate as normal hard-core processors. For example, Xilinx Microblaze usually runs under 400 MHz, while Intel and ARM processors can run at well over 1 GHz [25,26,27,28,29].

When users program these soft processor systems, they do not normally have to think in terms of the hardware but at a relatively high-level, and potentially get decent performance. Unfortunately, there are no soft processors optimized directly for image processing from vendors such as Intel (Altera) and Xilinx. Two soft processors developed specifically for image processing are, for example, IPPro [30] and a RISC-V soft processor [31]. These processors require fewer resources than Nios II and Microblaze.

### 2.3. Image Algebra and Pixel Level Abstractions

Image Algebra (IA) [32] is a mathematical theory concerned with the transformation and analysis of digital images at the whole image (rather than pixel) level. Its main goal is the establishment of a comprehensive and unifying theory of image transformations, image analysis and image understanding. Basic IA operations can be classified as: point operations, neighborhood operations and global operations.

In point operations (P2P), the same operation is applied at every input pixel position using only pixels at that position. Operations can be binary or unary; they include relational (e.g., ‘>’, ‘=’), arithmetic (e.g., ‘+’, ‘×’), and logical (e.g., ‘and’, ‘or’) operations. Normally one output pixel is generated for each corresponding input pixel position.

A neighborhood operation (N2P) is applied to each (potentially overlapping) region of an image. It is most common to use a 3 × 3 or 5 × 5 window. A new pixel value will be generated for each window position. The user specifies the matrix of weights for the window which is used in calculating the result value.

A global operation is a reduction operation that is applied to the whole image and produces a scalar (R2S) or a vector (R2V). For example, global maximum will produce one scalar value, whereas histogram will produce a 256-element vector (for standard grey level images).

### 2.4. FPGA-Based Image Processing

In embedded systems, FPGAs are powerful tools for accelerating image processing algorithms, especially for real-time embedded applications, where latency and power are important considerations. FPGAs can be embedded in a camera to directly provide pre-processed image streams. In this way, the sensor will provide an output data stream rather than merely a sequence of images [33]. FPGAs can achieve both data parallelism and task parallelism within many image processing tasks. Unfortunately, simply putting a PC-based algorithm onto an FPGA usually gives disappointing results [34]. In addition, many image processing algorithms have been optimized for scalar processors. Thus it is usually necessary to optimize the algorithm specifically for an FPGA before implementing.

There have typically been three approaches to implementing an image processing algorithm/system on FPGAs:

1. Custom hardware designed using Verilog HDL or VHDL and combined with the vendor’s IPs.
2. High-level synthesis tools used to convert a C-based representation of the algorithm to hardware.
3. Algorithm mapped onto a network of soft processors.

When users need to implement an algorithm on FPGAs using custom cores, they need to consider the memory mapping, architecture, and algorithmic optimizations. On the other hand, when users try to use soft processors to implement their complex algorithm, they will usually be limited by the poor single core performance on the one hand, and resource utilization of a multi-core architecture on the other. Thus, balancing programmability, resource utilization and performance is a key challenge for implementing algorithms on FPGAs.

### 2.5. Summary

Currently, HLS tools are the key to rapidly implementing FPGA-based image processing algorithms or systems. HLS tools can even accept different input languages, such as C/C++, Java, Python and LabVIEW. Users need to use Xilinx Vivado or Intel Quartus Prime to perform the integration. This stage usually requires detailed hardware knowledge.

**Figure 1:**
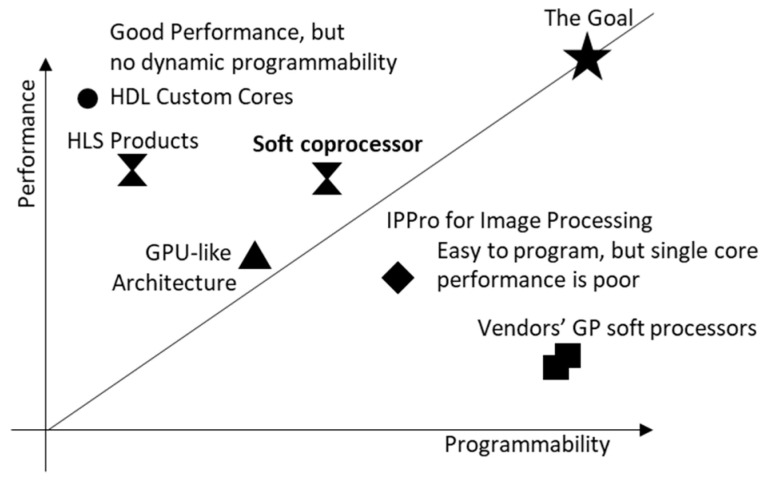
Qualitative representation of programmability vs. performance.

In terms of the efficiency of implementing image processing algorithms and systems on FPGAs, custom cores have better performance than soft processors, but require users to have detailed hardware knowledge to design efficient accelerators. Soft processors keep the high-level programming model, but single core performance is poor. Users need to use multiple soft processors in order to meet the performance requirements. Figure 1 indicates informally the programmability (ease of use) vs. performance (throughput) of the different approaches. Our goal is to move a step closer to achieving both performance and programmability at the same time. For suitable applications, our soft coprocessor approach seeks to have performance approaching HLS products and HDL custom cores, even if it is not as programmable or as general purpose as soft processors.

To address these challenges and problems, in this paper we present our approach—the soft coprocessor (SCP) approach. This aims to achieve performance closer to custom cores while providing users with a higher-level programming model than the current Vivado toolchain.

## 3. User’s View of the Soft Coprocessor Approach

### 3.1. The Concept of Soft Coprocessors

For FPGAs, performance and programmability are in conflict with each other. For specific applications such as image and signal processing, it is sometimes possible to present a higher level programming model which is less general purpose but can exploit common data access patterns. One of the first uses of coprocessors was in the early days of microprocessor design. For example, the Intel 8086 processor could use a separate 8087 coprocessor chip to increase the speed of floating-point calculations with which it was closely integrated [35]. A coprocessor does not have the usual overhead of the fetch-execute cycle which is a significant overhead for soft processors. We therefore propose the concept of soft coprocessors to attempt to obtain many of the benefits of an application-specific processor but with the efficiency of a coprocessor. All our soft coprocessors have the following basic properties:

- A standard interface for data transfer between soft coprocessors, allowing developers to add a soft coprocessor to a system without having to design custom I/O hardware.
- Each soft coprocessor can be parameterizable, allowing a degree of programmability and functional flexibility, but without requiring re-synthesis.
- The soft coprocessors should be able to interact with each other, and be formed into a DFG arrangement, to reduce communication and buffering overheads. This assumes a stream-based approach.
- Each soft coprocessor should be able to interact with the background control and communication system that manages the operation of the whole FPGA-based system.

FPGAs have a lot of computing resources but a more restrictive on-chip memory model. The efficient use of memory resources is crucial to system performance. Skilled developers can choose the optimal memory management approach from a range of possibilities. However, for application developers, it is difficult to fully exploit the limited on-chip memory resources using HLS tools. To provide optimized memory allocation for point, neighborhood and global operations, we provide three fundamental types of soft coprocessor based on the core Image Algebra (IA) operations.

### 3.2. Soft Coprocessors for Stream-Based Image Processing

Stream-based processing using on-chip memory is preferred where possible, since simultaneous access to off-chip memory by multiple coprocessors would be a bottleneck.

For a specific application domain such as image processing, we would like a set of SCPs which can be instantiated, and which cover the common domain operations. A good method to identify such a set is to find an existing algebra for the domain and build on the abstractions that have been identified and used at a mathematical level. In the case of image processing, we have chosen some of the core concepts of Image Algebra (IA).

### 3.3. Single Image Algebra-Based SCPs

We provide a built-in library of core SCPs which carry out the core operations of Image Algebra. There are four core classes of IA SCPs, plus a fifth type for compound operations:

(i) Point operations. We provide two types of SCP which apply a point function to every pair of pixels in the two streamed input images (or to each pixel and a scalar parameter), and generate an output pixel stream. The actual function applied is a parameter. The range of point functions include all the standard (integer) arithmetic, logical and relational functions. For example, a threshold operation would use the image-scalar SCP with the two parameters (≥, threshold value). Image stream pixels are held as 8-bit integers and intermediate values created during addition, subtraction and multiplication are held in higher precision as necessary.

(ii) Neighborhood operations. We provide an SCP for each common size of neighborhood (3 × 3, 5 × 5, etc.). The N × N matrix of weights is supplied as a parameter. A standard neighborhood operation has two functions: the point function which is applied pairwise to each pixel-weight pair in the window, then the reduction operation which reduces the N × N intermediate results to a single pixel result. For example, for a standard convolution, the two function parameters are (×, Ʃ). Using this type of SCP a range of common image processing functions are possible, such as dilation, erosion, convolution-based edge detection and image filtering.

For example, a simple dilation SCP on a binary image would be an instance of the 3 × 3 SCP with the kernel weights [1, 1, 1, 1, 1, 1, 1, 1, 1] and the functions (×, ‘or’) (effectively just a neighborhood OR). An erode SCP would have ‘and’ instead of ‘or’ as a parameter.

For some operations (perhaps involving image reduction), the window can step by more than one pixel: for example, in the convolution layer of a Convolutional Neural Network (CNN) [36]. This is achieved by having a stride parameter as part of the neighborhood operation SCP. The default stride is 1 × 1.

(iii) Global operations. We provide an SCP that performs a reduction operation on a streamed image. The result is a single value. The available reduction functions include Ʃ, |Ʃ|, max, min, count, and average. A second global SCP produces a vector as a result (typically used for finding the image histogram).

(iv) Block operations. Sometimes we need to divide an image into multiple smaller blocks and then apply the same algorithm to each block. For example, for the Histogram of Oriented Gradients (HOG) algorithm, we find a histogram of edge gradients for each block. Thus, we provide a block-based SCP that provides a neighborhood operation or other function, for each block separately.

(v) Common complex operations. Although the above basic SCPs can be chained together to perform a compound IA-based algorithm, in practice there are certain common patterns of operations which can be more efficiently implemented as a single operation. We therefore provide a number of pattern-specific SCPs. For example, edge-finding and morphological operations sometimes apply a window in several rotated orientations and have a final reduction stage to produce a single result. We provide a Cycle Neighborhood SCP that takes as its parameters the weight matrix, the number and angle of rotations, the two functions for the neighborhood operation, and the final reduction operation.

For example, suppose we want a complete Sobel edge detection operation using a single complex neighborhood SCP. We supply the kernel (the vertical one, say) and specify two orientations, with a rotation angle of 90^o^. The two neighborhood function parameters are “×” and “|Σ|” and the vector of kernel weights is [−1, 0, 1, −2, 0, 2, −1, 0, 1]. The final operation to combine the two window outputs (the vertical and horizontal edge strengths) is ‘+’. (Adding the absolute edge strengths is a common approximation to avoid squaring and adding).

### 3.4. Chaining Multiple SCPs in a Data Flow Graph

Multiple instances of the above generic SCPs can be chained together to implement a compound algebraic expression. The output stream of one SCP is fed directly as the input to the next without buffering the complete intermediate image or without involving the host processor. Synchronization is handled automatically by the SCP framework. This chaining can be represented by a simple Data Flow Graph (DFG).

For example, the above Sobel edge detector could have been created using two basic 3 × 3 neighborhood SCPs feeding their results into a third point SCP.

### 3.5. Skeleton SCPs for Function-Specific Coprocessors

Using generic SCPs is useful during the algorithm experimentation stage because the hardware does not need to be changed even if different functions are selected. However, once the algorithm is finalized, more efficient function-specific coprocessors for compound operations can be created. To make this convenient without requiring hardware knowledge, we provide a set of SCP skeletons. These are effectively hollow codings of the above four classes of SCP (point, neighborhood, global and block). The skeletons contain HLS code to manage the dataflow patterns of each type of operation. In this way, users need only to supply the core pixel-level function in the form of a simple C/C++ function. It is in this C function that the user specifies the arbitrarily complex operation. Users can code detailed optimizations, for example, by embedding constant kernel coefficients. An example that we will see later is an SCP specifically for a more efficient implementation of the Sobel edge detector.

A new SCP created using our skeletons will need to be synthesized the first time. Once it is added to the SCP library, it is available thereafter.

Function-specific SCPs are commonly used to replace a chain of SCPs, or they can replace a generic SCP with one that is optimized for the specific purpose. For example, a more efficient dilation SCP could be created using the 3 × 3 neighborhood skeleton and encoding a simple OR function which avoids the need to apply the redundant ×1 step.

Function-specific SCPs will be more area-efficient than their generic counterparts. Each generic SCP must retain the hardware for all the available functions, in case the user wishes to experiment with different functions during development, without resynthesis. Of course, the function-specific SCPs are not as functionally flexible. There are also several coding conventions which must be followed, for accessing the parameters. This is one of the necessary trade-offs when working with FPGAs.

**Figure 2:**
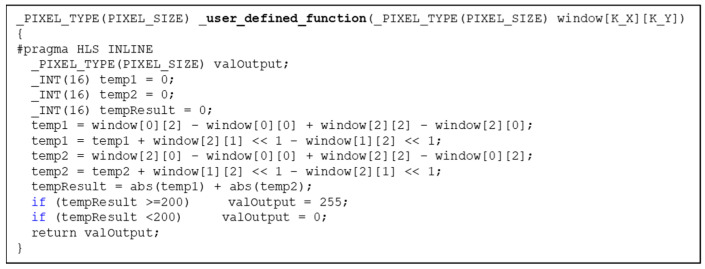
The core function for the Sobel operation when using a skeleton SCP.

We now provide an example of using a neighborhood skeleton SCP to implement a Sobel operation as a single and efficient function-specific SCP. The code of the Sobel function, including thresholding, is shown in Figure 2.

### 3.6. Generating SCP Configurations

We distinguish between the application program and the hardware configuration it runs on. To avoid frequent re-synthesis, our model is that a (pre-synthesized) configuration contains the set of SCPs which are available to the application developer. Provided the application makes use of only these SCPs, then changes to the application can be made without any re-synthesis. There are separate tools for defining both the configurations and the application.

**Figure 3:**
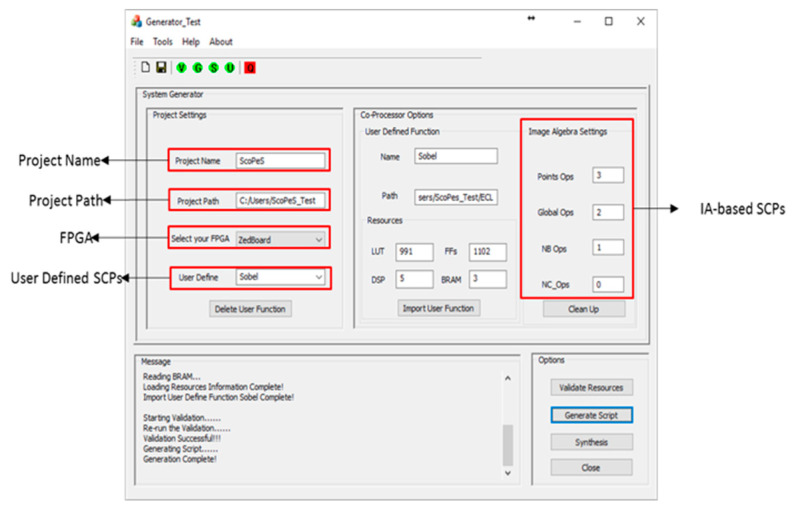
The GUI for creating a new project configuration.

To speed up the process of getting a runnable FPGA configuration, our SCoPeS environment maintains a library of FPGA configurations which contain different mixes of SCPs from the SCP library. The need for this arises because the developer may not know in advance exactly how many instances of each type of SCP will be needed. If the Configuration library does not have the necessary mix for the current project, then we provide a tool which enables the user to create a new SCP configuration. The user can specify the number of each class of SCP, and the Hardware Configuration Generator (HGC) tool will then generate the complete FPGA bitstream, and add it to the Configuration library, as shown in Figure 3. Obviously, the required hardware resources of the defined configuration must be able to fit on to the target FPGA.

### 3.7. Text-Based DFG Code Generator (TCG)

Normally, users could use the default Xilinx SDK to program the Zynq-based hardware platform in baremetel mode or use PetaLinux+Xilinx SDK to build a Linux-based application. In this stage, there is no hardware level design; normally users can develop their application in C/C++. Users need to use the HLS-exported driver to create their own initialization function, set all the parameters individually, and invoke them for execution. We use the AXI4-Stream Interconnect for connecting all our coprocessors (see later) to match the user-supplied DFG. This textual DFG specifies the coprocessor instances, their parameters, and their interconnection channels. Our Textual Code Generator (TCG) tool takes the text representation of the DFG and generates the executable C code for the Xilinx SDK. This simplifies and speeds up the development of the final application.

**Figure 4:**
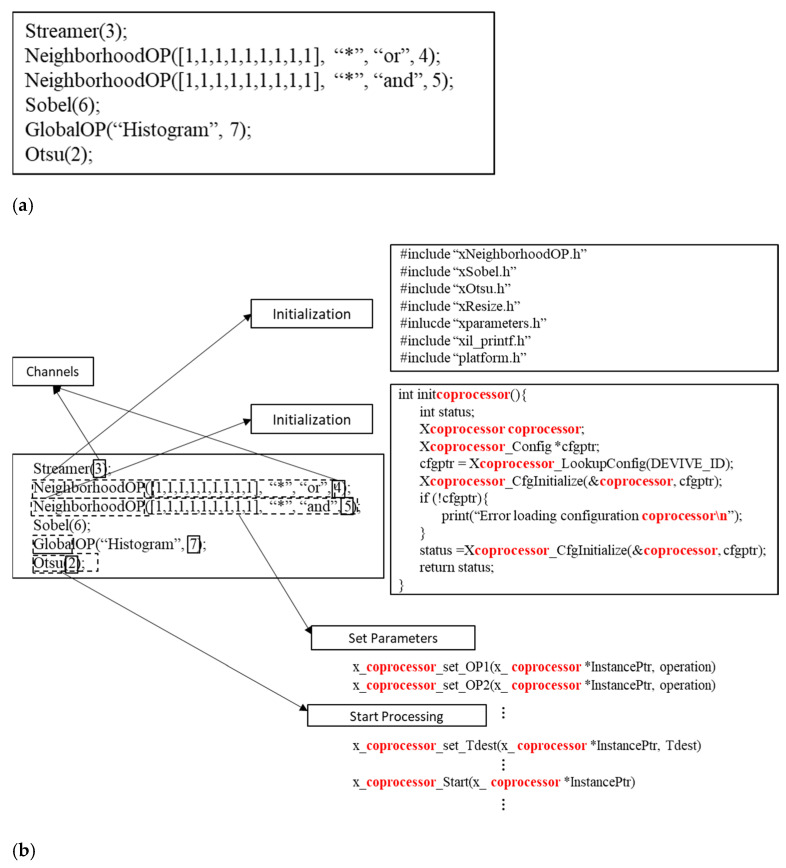
(**a**) Example of the textual description of a DFG for Otsu after an Open operation. (**b**) Example of the code generated by the TCG tool from the DFG in Figure 4a.

As an example, Figure 4a shows the developer’s code (the textual DFG) for an automatic thresholding system using the Otsu method (assuming we have already written the final Otsu SCP to select and apply the threshold using our skeletons) after an Open operation. The ‘Streamer’ is a block which is directly connected to the camera, and which generates a stream with all the parameters and the image data. We set the ‘Streamer’ output channel to channel 3. We then do the dilation and erosion through neighborhood operations. After that, we do the edge detection, histogram finding and Otsu thresholding. The result image stream is returned through channel 2. Figure 4b outlines the generated Xilinx SDK useable code from the DFG in Figure 4a.

In Figure 4b, the first block on the right is the output from the first pass of our TCG tool through the text description, generating all the necessary header files based on the names of the functions. The second block shows the generated initialization functions. The main body of the program is then generated based on the text-based DFG.

### 3.8. Using the SCoPeS Development Environment

Our SCoPeS development environment includes the tools necessary to build an application using the SCP library, as mentioned above. It is currently a prototype IDE. The typical design flow for a new project/application is thus as follows:

1. Decompose the desired algorithms into IA expressions.
2. Select (or create) a suitable configuration from the Configuration library. (We can select a different one later if we run out of instances of a certain type of SCP).
3. Define each algorithm as a Data Flow Graph (DFG) and use the TCG tool to set up the system defined by the DCG.
4. Experiment with the system, until the functions and parameters are finalized.
5. If necessary, design function-specific coprocessors to replace some of the IA-based SCPs selected in step 2.
6. If step 5 was utilized, import the function-specific coprocessors into the system and resynthesize the system configuration.

## 4. Architectures and Implementations of Coprocessors

In this section, we discuss some key implementation aspects of the SCP approach, including the architecture for single IA-based SCPs, hardware skeletons and hardware configurations.

### 4.1. SCP Architectures for Image Algebra Operation Types

**Figure 5:**
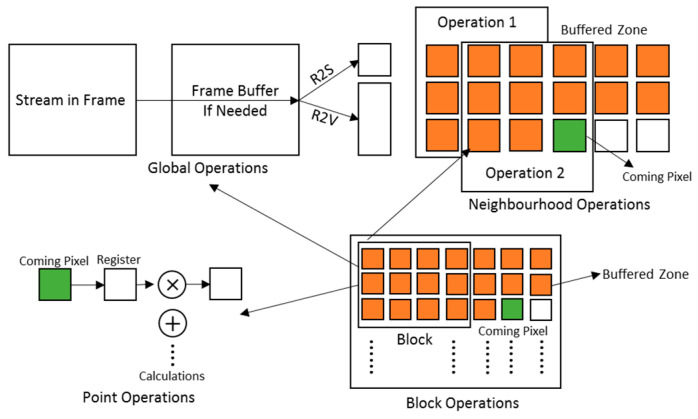
Data flow and buffering for the four different operation types (clockwise: Global, Neighborhood, Block and Point operations).

When implementing the SCPs on FPGAs, the use of the internal memory depends on the type of operation. Point operations usually do not need image buffers; neighborhood operations require line buffers to hold the relevant pixels within the window depending on the size of the kernel. Some global operations do not require any buffering; however, some function-specific global SCPs may need a whole frame buffer to hold the frame until the frame has been processed, such as Otsu adaptive thresholding [30]. When creating an instance of one type of SCP, the optimized data handling then comes for free. Figure 5 shows how we handle the data flow and buffering in different types of SCP. Since we are using HLS to implement these SCPs, details of the architectures are hidden from us, and we only have control over the data flow and buffering.

The point operation SCP reads the next pixel from the input stream and performs the calculation before pushing the result to the output stream. With pipelining, one pixel is output every clock cycle.

In the neighborhood operation SCP (e.g., convolution), the example architecture of a generic 3 × 3 neighborhood operation is shown in Figure 5. As the streamed pixels arrive, we use a BRAM-based line buffer to hold two lines and two pixels. When the third pixel of the third line arrives, we have the whole window ready for a neighborhood operation to produce one single output pixel. Then, we increment the window position, read one more pixel, and perform the next neighborhood operation. The neighborhood calculation in our generic operator is divided into two stages. In the first stage, for each position in the window, each image pixel in the window is combined pairwise with the corresponding value in the kernel (the matrix of window weights supplied by the user). These intermediate results are then reduced in the second stage. (For convolution, this would be an accumulation operation.)

As a global operation can reduce a streamed input image to either a scalar result or a vector result, two versions of global SCP, R2S and R2V, are available. Sometimes the result of a global operation is subsequently used to process the same image (e.g., to threshold an image based on its average pixel value). In this case, it will be necessary to buffer the whole input image in an image buffer. Thus, in the architecture for a global operation SCP (Figure 5), when a streamed image comes from a camera, another SCP, or from a file, users can decide if they need a built-in frame buffer before pushing the result pixel. During the buffering or streaming of the input frame, the calculation for the global operation can be performed at the same time, since the global SCPs are fully pipelined. Supported operations include Min, Max, Σ, |Σ|, Count and Global Average. An image histogram can be obtained by selecting the R2V SCP and specifying the address in BRAM where the vector will be stored so that subsequent SCPs can access the result directly. However, when internal memory allocation such as a frame buffer is needed, re-synthesis may be required.

The block operation can be regarded as a special neighborhood operation that operates on a stream of blocks. This requires an outer level of processing to extract blocks in order, and to stream each block to the neighborhood operation. For each block, we can perform any neighborhood-based operation. When performing a neighborhood operation (e.g., 3 × 3) on a block, we must allow for the edge effect at block boundaries. Therefore, the block buffer is one column larger (for a 3 × 3 operation) than the original block (see Figure 5). Moreover, the buffering hardware will handle any block stride length dynamically in SCPs, as it is sometimes useful to experiment with different block strides at runtime.

Complex SCPs which perform a neighborhood operation with a kernel in multiple orientations avoid the need to replicate the line buffer. Using the complex neighborhood SCP, and supplying the appropriate kernel plus the rotation parameters, we can execute these operations in a single pass of the stream. This solution uses only a single line buffer.

### 4.2. Communication between Coprocessors

To allow users to change the DFG interconnections between SCPs without re-synthesis, we use AXI4-Stream Interconnect (a Xilinx provided IP core) to connect SCPs instead of using naïve FIFOs. Each SCP has a TDEST input to indicate where its output stream goes in the AXI4-Stream Interconnect system.

When there are many SCPs in the application, there will be many parameters to be sent to the various SCPs, so it is crucial to find an efficient way of distributing these parameters. We also would like parameter distribution to be dynamic (in the sense that they can be changed while the program is running). Our solution is to send the parameters as part of the header package for every new frame. It would be possible to send them using the ARM processor through the AXI bus using the AXI-Lite interface [32] by enabling the data stream [33], but the ARM would have to work sequentially in sending all the parameters every frame, which is time-consuming when there are many SCPs involved. This is why our approach is to group the command and data together by appending the parameters to the front of each frame in the image data stream.

**Figure 6:**
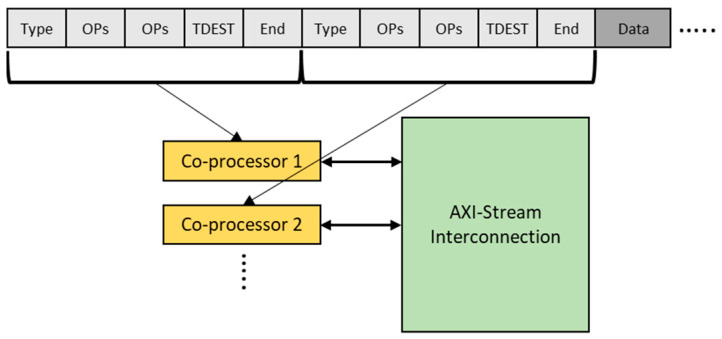
Stream-based parameter distribution.

The parameter stream is illustrated in Figure 6. The parameter stream comprises, for each SCP, the ID of the SCP, its various parameters, and the output channel (TDEST). Because we fix the entry point of the system to be the streamer, in this particular case we only need to define the output channel of each SCP. (More generally, of course, both the input and output channels would be defined). Each SCP receives the complete parameter stream for all SCPs; it extracts only those parameters relevant to it, passes on the parameter stream to the output channel (the next SCP), and then starts processing the image data which follows the parameter section.

### 4.3. Coding SCPs behind the Scenes

We created the Image Algebra-based soft coprocessors using Xilinx Vivado HLS. For interoperability of SCPs, the way of interfacing any coprocessor to the rest of the system is always the same.

**Figure 7:**
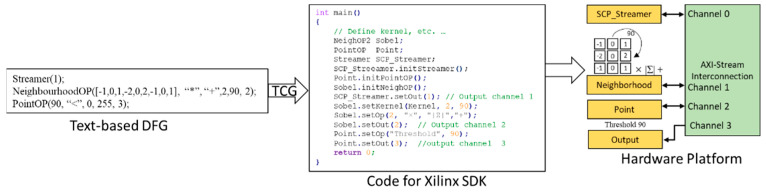
From text-based DFG to hardware platform through Xilinx SDK.

When the developer introduces a new SCP instance in the textual DFG description, behind the scenes, one of the free instances of the SCP will be acquired from those still available in the user-selected configuration. The parameters in the DFG are used by the TCG tool to generate and set the various properties of the SCP in an object-oriented fashion. Code is also generated to form the connections via the channels in the AXI4-Stream Interconnection scheme described above. This code is for the Xilinx SDK after the hardware platform has already been defined and synthesized. For example, Figure 7 shows the TCG-generated code for the Xilinx SDK to set up a complex SCP (of type NeighOP2) followed by a thresholding SCP (of type PointOP) for the Sobel operation outlined previously, based on a two-step rotating kernel.

When implementing designs using Xilinx Vivado HLS, directive settings (or pragmas) can have a significant effect on hardware utilization and performance. Optimization using well-designed directives can be several times more effective than an unoptimized design. Mastering these directive settings takes a lot of time and requires a deeper understanding of how the hardware works. We therefore developed our own internal library of reusable macros and reserved variables which we used to simplify and standardize the HLS coding of all the IA SCPs. These macros are also available to the developer when creating skeleton-based function-specific SCPs and when writing the low-level C function. This library is not normally required to be visible to the developer, but we mention it as a valuable approach to simplify the retargeting of our HLS coding of SCPs and skeletons to other types of FPGA. This internal library includes:

- Interface settings;
- Pipelining directives;
- Buffer settings;
- Special data types and hardware-level signal handling.

## 5. Evaluation and Comparisons

In this section, we present some details of the performance and hardware utilization of the SCPs. We use the Xilinx Zedboard with an I2C OV7670 camera module as the test platform. The OV7670 camera can produce a 640 × 480 8-bit greyscale video stream and can be connected to the Zedboard. The Zedboard is equipped with an XC-7Z020 FPGA, which has programmable logic (PL) and an ARM processor. We use the Xilinx Zedboard to implement our designs and evaluate two different versions of our IA-based SCPs: the Minimum Area mode and Maximum Performance mode (these have to be separately synthesized). We compare example operations using SCPs with equivalent implementations using the image processing soft processor, IPPro. Finally, we also compare a generic (complex) single SCP formulation of a Sobel operator with an equivalent function-specific SCP created using a neighborhood skeleton SCP.

### Performance and Hardware Utilization

Table 1 shows the SCPs’ hardware utilization and performance (in frames per second) on a Virtex FGPA running at 150MHz in Minimum Area mode. This is compared with the utilization and performance of the soft processor-based solution using a multi-core IPPro. The comparison is for four basic SCP operations (point, neighborhood, complex and global). Table 2 shows the equivalent figures using Maximum Performance mode for the SCPs. In both cases, the image size is 512 × 512 and in the neighborhood operation SCP, the kernel is a 3 × 3 matrix.

The first observation is on the difference between Minimum Area mode and Maximum Performance mode. Maximum Performance mode is roughly three times as fast, but takes twice as much area, as Minimum Area mode. However, in practice there may be no advantage in being able to process at nearly 400FPS, and so the Minimum Area mode is often to be preferred.

To make comparison with various IPPro configurations easier, Table 3 shows the normalized ratios of performance and resources (to one decimal place) based on the data in Table 1 and Table 2 (first for Minimum Area mode and then for Maximum Performance mode). Note that a value greater than 1 in the IPPro rows indicates the degree to which IPPro is worse than SCP. Thus in Maximum Performance mode, SCPs process 4.6 times faster than IPPros in point operations and 7.3 times faster in neighborhood operations, while using less hardware than IPPro. This is partly because the IPPro has to go through the standard fetch-execute cycle. In Minimum Area mode, SCP performance is a little faster than IPPro, yet uses only 20% of the resources (apart from DSPs) as Table 3 shows.

To illustrate the benefit of using a function-specific SCP, we choose Sobel for our final comparison. We compare the generic complex SCP with a function-specific SCP in doing a Sobel operation in Table 4.

Interestingly, the generic SCP approach and the function-specific SCP have very similar performance (around 125FPS for a 640 × 480 video stream). However, the skeleton approach is clearly much more area efficient (by a factor of approximately 10), because it removes all the unused function logic which is part of the generic SCP.

## 6. Conclusions

In this paper, we have presented several concepts and tools which are intended to make it easier for application developers to design FPGA-based image and video processing systems while designing at a high-level. By high-level, we do not mean merely using the syntax of a high-level language; we mean designing systems with no, or as little as possible, hardware knowledge. Where it becomes necessary to drop down into hardware design, we have introduced approaches and customizable components intended to abstract away many of the hardware-aware details.

Our main specific conclusions are as follows:

1. We propose the concept of soft coprocessors, which are single-instruction processors that can be parameterized to support a range of different functions. SCPs can be assembled into a DFG for efficient stream-based processing.
2. The SCPs allow users to conveniently design and experiment with an image processing application by chaining SCPs together. We use AXI4-Stream Interconnect to connect all the SCPs in the system in a way that reflects the algorithm’s Data Flow Graph (DFG). In this way, we provide users with a flexible system that can be programmed as a textual DFG. Users do not need to re-synthesize when they change the DFG.
3. We provide reusable SCP skeletons to allow developers to create efficient function-specific coprocessors without needing to know (much) about hardware structures.
4. We have provided a set of generator tools which comprise the SCoPeS environment—a prototype IDE to support the SCP concept.
5. Overall, we conclude that the soft coprocessor approach has the potential to deliver better performance than the soft processor approach, and can improve programmability over dedicated HDL cores for domain-specific applications while achieving competitive real-time performance and utilization.

However, our work also has the following main limitations:

1. Our current work is designed only for image and video processing development, and is not a general-purpose tool. However, as a general rule, the coprocessor approach is suited to any application area that has an associated under-pinning algebra.
2. Our implementation currently only supports relatively simple DFGs.
3. Our tools do not yet support image partitioning for greater parallelism, which can be a useful additional technique for accelerating image processing applications. Updating our tools to include this option of a multi-core approach is a promising future development.

## Figures and Tables

**Table 1:** Comparison between SCP (in Minimum Area mode) and IPPRO in utilization and performance.

**SCPs**	**FFs**	**LUTs**	**BRAMs**	**DSPs**	**FPS**
Point	1659	2015	0	3	186
Neighborhood Basic	1104	1404	5	9	127
Neighborhood Complex	4963	7141	5	72	125
Global	622	998	0	0	189
**IPPro** [30]	**FFs**	**LUTs**	**BRAMs**	**DSPs**	**FPS**
Point (8 core)	12,279	10,941	18.5	8	120
Neighborhood Basic (6 core)	13,202	11,826	32.5	6	76

**Table 2:** SCP utilization and performance (in Maximum Performance mode).

SCPs	FFs	LUTs	BRAMs	DSPs	FPS
Point	3346	2965	0	3	556
Neighborhood Basic	2309	1963	5	9	380
Neighborhood Complex	9862	12,368	5	72	374
Global	1432	1353	0	0	568

**Table 3:** Ratios for SCP to IPPro for performance and utilization (>1 is worse).

**Min Area**	**Operation**	**Performance**		**Utilization (>1 Is Worse)**			
		**Freq**	**FPS**	**FFs**	**LUTs**	**BRAMs**	**DSPs**
Point	SCP	150 MHz	1	1	1	1	1
	IPPro (8 core)	150 MHz	1.5	7.4	5.4	---	2.7
Neighborhood	SCP	150 MHz	1	1	1	1	1
	IPPro (6 core)	150 MHz	2.4	8.0	5.9	---	2.0
**Max Performance**	**Operation**	**Performance**		**Utilization**			
		**Freq**	**FPS**	**FFs**	**LUTs**	**BRAMs**	**DSPs**
Point	SCP	150 MHz	1	1	1	1	1
	IPPro (8 core)	150 MHz	4.6	3.7	3.7	---	2.7
Neighborhood	SCP	150 MHz	1	1	1	1	1
	IPPro (6 core)	150 MHz	7.3	5.7	6.0	---	0.7

**Table 4:** Comparison between a generic and a function-specific SCP.

SCP Type	FFs	LUTs	BRAMs	DSPs	FPS
Generic	9862	12,368	5	72	125
Function-specific	932	1107	2	3	128

## Data Availability

Not applicable.

## References

[1] Hong D., Han Z., Yao J., Gao L., Zhang B., Plaza A., Chanussot J. (2021). SpectralFormer: Rethinking hyperspectral image classification with transformers. IEEE Trans. Geosci. Remote Sens..

[2] Wu T., Yang Z. (2021). Animal tumor medical image analysis based on image processing techniques and embedded system. Microprocess. Microsyst..

[3] Khasanova A., Makhmutova A., Anikin I. Image Denoising for Video Surveillance Cameras Based on Deep Learning Techniques. Proceedings of the 2021 International Conference on Industrial Engineering, Applications and Manufacturing (ICIEAM).

[4] Kalinowska K., Wojnowski W., Tobiszewski M. (2021). Smartphones as tools for equitable food quality assessment. Trends Food Sci. Technol..

[5] Nguyen M.T., Truong L.H., Le T.T. (2021). Video surveillance processing algorithms utilizing artificial intelligent (AI) for unmanned autonomous vehicles (UAVs). MethodsX.

[6] Aslan S., Güdükbay U., Töreyin B.U., Çetin A.E. (2019). Deep convolutional generative adversarial networks based flame detection in video. arXiv.

[7] Arvin R., Khattak A.J., Qi H. (2021). Safety critical event prediction through unified analysis of driver and vehicle volatilities: Application of deep learning methods. Accid. Anal. Prev..

[8] Siska J., Jaeschke T., Wagner J., Pohl N. FPGA-Accelerated Multispectral Ultra-High Resolution SAR-Imaging with Wideband FMCW Radars. Proceedings of the 2019 IEEE Radio and Wireless Symposium (RWS).

[9] Attaran N., Puranik A., Brooks J., Mohsenin T. (2018). Embedded low-power processor for personalized stress detection. IEEE Trans. Circuits Syst. II Express Briefs.

[10] Chen X., Tan H., Chen Y., He B., Wong W.F., Chen D. ThunderGP: HLS-based graph processing framework on fpgas. Proceedings of the 2021 ACM/SIGDA International Symposium on Field-Programmable Gate Arrays.

[11] Yuan H., Ding D., Fan Z., Sun Z. A Real-Time Image Processing Hardware Acceleration Method based on FPGA. Proceedings of the 2021 6th International Conference on Computational Intelligence and Applications (ICCIA).

[12] Xiao Z., Chamberlain R.D., Cabrera A.M. HLS Portability from Intel to Xilinx: A Case Study. Proceedings of the 2021 IEEE High Performance Extreme Computing Conference (HPEC).

[13] Winterstein F., Bayliss S., Constantinides G.A. High-level synthesis of dynamic data structures: A case study using Vivado HLS. Proceedings of the 2013 International Conference on Field-Programmable Technology (FPT).

[14] Liu S., Lau F.C., Schafer B.C. Accelerating FPGA prototyping through predictive model-based HLS design space exploration. Proceedings of the 2019 56th ACM/IEEE Design Automation Conference (DAC).

[15] Coussy P., Gajski D.D., Meredith M., Takach A. (2009). An introduction to high-level synthesis. IEEE Des. Test Comput..

[16] O’Loughlin D., Coffey A., Callaly F., Lyons D., Morgan F. Xilinx vivado high level synthesis: Case studies. Proceedings of the 25th IET Irish Signals & Systems Conference 2014 and 2014 China-Ireland International Conference on Information and Communications Technologies (ISSC 2014/CIICT 2014).

[17] Gaide B., Gaitonde D., Ravishankar C., Bauer T. Xilinx adaptive compute acceleration platform: VersalTM architecture. Proceedings of the 2019 ACM/SIGDA International Symposium on Field-Programmable Gate Arrays.

[18] Chatarasi P., Neuendorffer S., Bayliss S., Vissers K., Sarkar V. Vyasa: A high-performance vectorizing compiler for tensor convolutions on the Xilinx AI Engine. Proceedings of the 2020 IEEE High Performance Extreme Computing Conference (HPEC).

[19] Kathail V., Hwang J., Sun W., Chobe Y., Shui T., Carrillo J. SDSoC: A higher-level programming environment for Zynq SoC and Ultrascale+ MPSoC. Proceedings of the 2016 ACM/SIGDA International Symposium on Field-Programmable Gate Arrays.

[20] Domingo R., Salvador R., Fabelo H., Madronal D., Ortega S., Lazcano R., Juárez E., Callicó G., Sanz C. High-level design using Intel FPGA OpenCL: A hyperspectral imaging spatial-spectral classifier. Proceedings of the 2017 12th International Symposium on Reconfigurable Communication-Centric Systems-on-Chip (ReCoSoC).

[21] Canis A., Choi J., Fort B., Syrowik B., Lian R.L., Chen Y.T., Hsiao H., Goeders J., Brown S., Anderson J. (2016). Legup high-level synthesis. FPGAs for Software Programmers.

[22] Wakabayashi K. CyberWorkBench: Integrated design environment based on C-based behavior synthesis and verification. Proceedings of the 2005 IEEE VLSI-TSA International Symposium on VLSI Design, Automation and Test, (VLSI-TSA-DAT).

[23] Guo L., Chi Y., Wang J., Lau J., Qiao W., Ustun E., Zhang Z., Cong J. AutoBridge: Coupling Coarse-Grained Floorplanning and Pipelining for High-Frequency HLS Design on Multi-Die FPGAs. Proceedings of the 2021 ACM/SIGDA International Symposium on Field-Programmable Gate Arrays.

[24] Noronha D.H., Salehpour B., Wilton S.J. LeFlow: Enabling flexible FPGA high-level synthesis of tensorflow deep neural networks. Proceedings of the FSP Workshop 2018, Fifth International Workshop on FPGAs for Software Programmers.

[25] Hebbar SR R., Milenković A. SPEC CPU2017: Performance, event, and energy characterization on the core i7–8700K. Proceedings of the 2019 ACM/SPEC International Conference on Performance Engineering.

[26] Beutel J., Trüb R., Forno R.D., Wegmann M., Gsell T., Jacob R., Keller M., Sutton F., Thiele L. The dual processor platform architecture: Demo abstract. Proceedings of the 18th International Conference on Information Processing in Sensor Networks.

[27] Bellemou A., Benblidia N., Anane M., Issad M. (2019). Microblaze-based multiprocessor embedded cryptosystem on FPGA for elliptic curve scalar multiplication over Fp. J. Circuits Syst. Comput..

[28] Shamseldin A., Soubra H., ElNabawy R. Performance of DSP operations implemented using a soft microprocessor: A case study based on Nios II. Proceedings of the 2021 International Conference on Microelectronics (ICM).

[29] Mplemenos G.G., Papaefstathiou I. Mplem: An 80-processor fpga based multiprocessor system. Proceedings of the 2008 16th International Symposium on Field-Programmable Custom Computing Machines.

[30] Siddiqui F., Amiri S., Minhas U.I., Deng T., Woods R., Rafferty K., Crookes D. (2019). Fpga-based processor acceleration for image processing applications. J. Imaging.

[31] Kimura Y., Kikuchi T., Ootsu K., Yokota T. Proposal of Scalable Vector Extension for Embedded RISC-V Soft-Core Processor. Proceedings of the 2019 Seventh International Symposium on Computing and Networking Workshops (CANDARW).

[32] Wilson J.N., Ritter G.X. (2000). Handbook of Computer Vision Algorithms in Image Algebra.

[33] Liu G., Luo Q., Liu B., Lu B., Guo P. Embedded intelligent camera algorithm based on hardware IP. Proceedings of the Tenth International Symposium on Precision Engineering Measurements and Instrumentation.

[34] Bailey D.G. (2019). Image processing using FPGAs. J. Imaging.

[35] Palmer J.F. The Intel 8087 numeric data processor. Proceedings of the National Computer Conference.

[36] Li Z., Yang W., Peng S., Liu F. (2004). A survey of convolutional neural networks: Analysis, applications, and prospects. arXiv.

